# Spontaneous Rupture of the Urinary Bladder Following a Night of Binge Drinking

**DOI:** 10.7759/cureus.117333

**Published:** 2026-10-01

**Authors:** Mehdi Nabavi, Mahmoud Shafii

**Affiliations:** 1 Urology, Hywel Dda University Health Board, Carmarthen, GBR

**Keywords:** acute surgical abdomen, binge drinking, intraperitoneal bladder rupture, spontaneous rupture of urinary bladder, unable to void

## Abstract

Spontaneous rupture of the urinary bladder is a rare but potentially life‑threatening condition that often presents with non‑specific symptoms, leading to delayed diagnosis. We report a case of spontaneous intraperitoneal bladder rupture in a man in his 40s following acute bladder overdistension after binge alcohol consumption. The patient presented with lower abdominal pain, inability to pass urine, and elevated creatinine and lactate levels. Imaging revealed free intraperitoneal fluid and an intravesical haematoma. With clinical suspicion of spontaneous bladder rupture, an emergency laparotomy was performed, confirming a 15 cm rupture of the bladder dome. Primary two‑layer repair with drain placement was undertaken, and the patient made a full recovery, with an intact bladder demonstrated on post‑operative cystogram and successful catheter‑free voiding by day 18. This case highlights the importance of early recognition of spontaneous bladder rupture in patients presenting with acute abdomen, particularly following alcohol intake, and underscores key diagnostic pitfalls, including unreliable bladder scanner measurements and pseudo‑renal failure due to autodialysis. Prompt surgical intervention remains essential for intraperitoneal ruptures. Clinicians should also consider the potential contribution of bladder‑affecting medications, such as antimuscarinics, in precipitating rupture.

## Introduction

Spontaneous rupture of the urinary bladder (SRUB) is a rare but potentially life‑threatening condition, with an incidence reported between 1:126,000 and 1:50,000 in the literature [1,2]. Mortality has historically been reported as high as 47% [3], although a recent systematic review suggests a rate closer to 15% [4].

Bladder ruptures are broadly classified as intraperitoneal or extraperitoneal. Intraperitoneal ruptures typically occur at the bladder dome, allowing urine to spill into the peritoneal cavity and causing chemical peritonitis, which necessitates urgent surgical repair. Extraperitoneal ruptures are more commonly associated with pelvic trauma and are frequently managed conservatively [5].

Bladder rupture most often results from blunt trauma or iatrogenic injury, whereas SRUB usually occurs in patients with predisposing factors that weaken bladder integrity, such as previous pelvic radiation, alcohol intoxication, post‑vaginal delivery, and infection or inflammation [4].

Alcohol intoxication is among the most frequently reported risk factors for SRUB, as binge drinking can lead to bladder overdistension and impaired sensation of fullness. Moreover, alcohol’s effects on consciousness and pain perception may reduce awareness of discomfort from urinary retention or minor trauma, which the patient may not recall [6].

Clinically, SRUB often presents with non‑specific features such as abdominal pain, abdominal distension, difficulty voiding, nausea, vomiting, and visible haematuria, making misdiagnosis common. Timely diagnosis relies on a high index of suspicion and imaging with cystography, CT cystography, or surgical exploration [4,7].

## Case presentation

A man in his 40s presented to our emergency department with lower abdominal pain and inability to pass urine at around midnight. Observations were stable. He had been unable to pass urine since the morning and initially experienced vague abdominal pain, 2-3/10 (0-10 Scale of Pain Severity), which progressively worsened to 10/10 by the time of presentation. There was no history of fever, trauma, nausea, vomiting, diarrhoea, constipation, or previous urinary symptoms.

The only relevant history was binge drinking the night prior, during which he consumed seven to eight pints of beer (3.9-4.5 L) and went to sleep without voiding. His past medical history included depression and hyperhidrosis, treated with mirtazapine and oxybutynin.

On examination, the abdomen was distended with mild generalized tenderness, worse in the suprapubic region, without guarding or rebound tenderness. Bowel sounds were present.

Investigations

A bladder scan showed 230 mL urine, which was inconsistent with the initial impression of acute urinary retention.

Laboratory investigations demonstrated elevated creatinine of 244 µmol/L (reference range: 58-110 µmol/L), mildly raised inflammatory markers with a white cell count of 12.5 × 10⁹/L (reference range: 4-11 × 10⁹/L) and C-reactive protein of 5 mg/L (reference range: <5 mg/L), and an elevated lactate level of 3.9 mmol/L (reference range: 0.5-1.6 mmol/L).

CT abdomen and pelvis with intravenous contrast showed a large volume of free intraperitoneal fluid with a hyperdense abnormality in the urinary bladder that could represent a haematoma (Figure 1).

**Figure 1:**
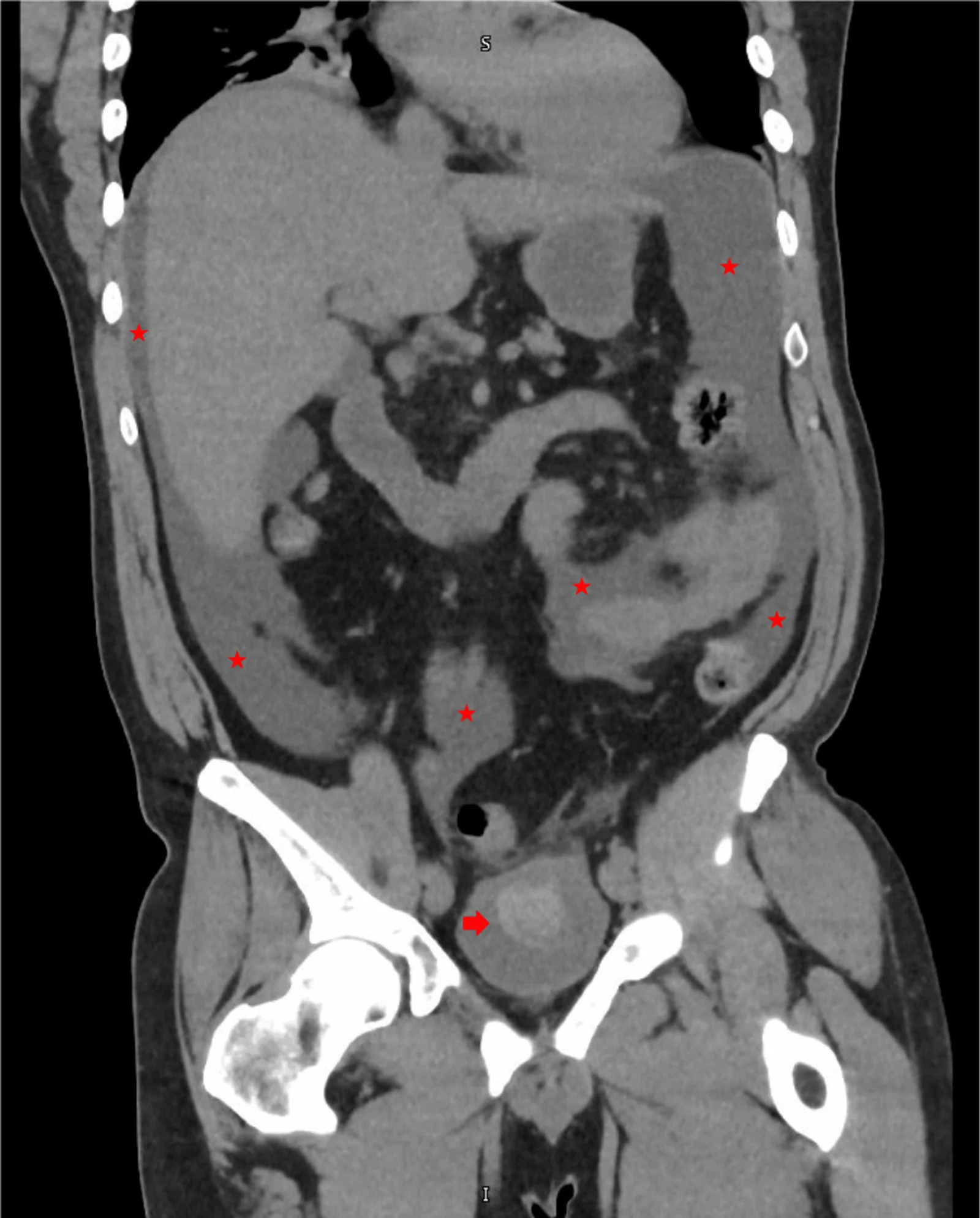
Contrast-Enhanced CT Scan of the Abdomen and Pelvis. Coronal section demonstrating a hyperdense abnormality within the urinary bladder (arrow) and intraperitoneal free fluid within the abdomen (asterisks).

Management

Catheterization using a two-way catheter drained 300 mL of bloody urine with clots, which provided significant symptomatic relief.

Following discussion with the on-call urology consultant, spontaneous bladder rupture was suspected. Broad-spectrum antibiotics (piperacillin-tazobactam) and intravenous fluids were commenced, and the patient was scheduled for emergency laparotomy.

Lower midline laparotomy revealed an intraperitoneal rupture extending from the left anterior bladder dome to the lower posterior bladder wall, 15 cm in length. Approximately 1.5 L of urinary extravasation was suctioned from the peritoneal cavity.

The bladder was closed in two layers with polyglactin 910 sutures, a 20 Fr Roberts drain was left in the pelvis, and the wound was closed in layers. A three-way indwelling urethral catheter was inserted.

Outcome and follow-up

The patient was admitted to the ward post-operatively for seven days and showed gradual improvement without complications. The drain was removed on post-operative day four when the output was less than 50mL per day. He was discharged with the urinary catheter in situ and scheduled for a cystogram two weeks later.

The cystogram demonstrated an intact bladder without contrast leakage or vesicoureteral reflux (Figure 2).

**Figure 2:**
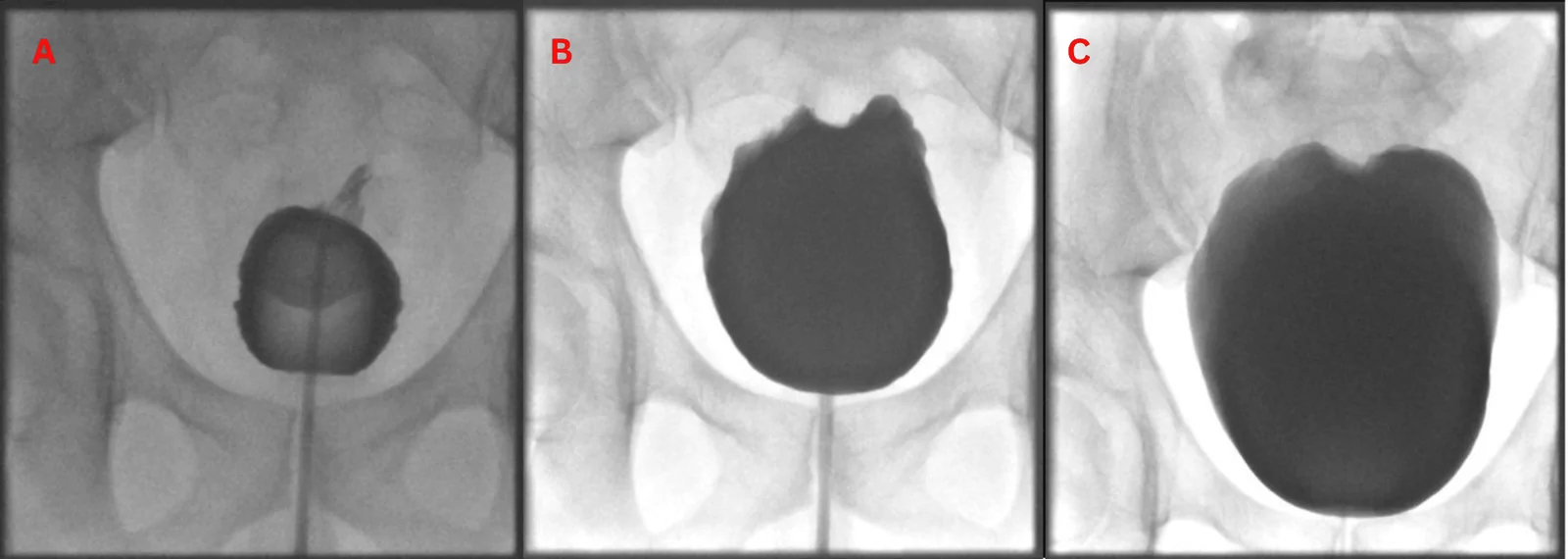
Cystogram. (A) Initial filling of the urinary bladder with contrast. (B) Progressive bladder distension with contrast. (C) Complete bladder filling without evidence of contrast extravasation or vesicoureteral reflux.

The patient underwent a successful trial without catheter on post-operative day 18 and returned to normal daily activities without complaints.

He was reviewed in the outpatient clinic at three months postoperatively. He remained asymptomatic, with a post-void bladder scan of 5 mL, and was subsequently discharged from urology follow-up.

## Discussion

SRUB is rare and often diagnosed late because of its non‑specific presentation. A systematic review reported that only 36% of cases were identified prior to intervention, with most initially misdiagnosed as gastrointestinal perforation; delayed diagnosis can lead to peritonitis, sepsis, and renal failure [7].

This case illustrates how acute bladder overdistension after binge drinking can precipitate intraperitoneal rupture, and how alcohol‑induced alterations in consciousness and pain perception may contribute to unrecognized or unremembered minor trauma. The patient’s use of oxybutynin, an antimuscarinic agent, may have facilitated prolonged bladder overdistension by reducing detrusor contractility and increasing bladder capacity. Oxybutynin can also delay the desire to void by the same mechanism through which it reduces lower urinary tract symptoms in patients with overactive bladder syndrome [8].

The elevated creatinine most likely reflected autodialysis across the peritoneal membrane rather than intrinsic renal failure. Extravasated urine within the peritoneal cavity creates a reservoir of creatinine‑rich fluid, allowing passive diffusion of solutes back into the circulation and producing a picture of pseudo‑acute kidney injury despite preserved renal function [4]. Normalization of renal parameters following surgical repair and drainage supports this mechanism (Table 1).

**Table 1:** Renal Function Trends Pre‑ and Post‑operatively. Creatinine reference range: 58-110 µmol/L eGFR: estimated glomerular filtration rate

Parameter	At presentation	Post-operative day 2
Creatinine	244 µmol/L	81 µmol/L
eGFR	25 mL/min/1.73m^2^	90 mL/min/1.73m^2^

This case also highlights a diagnostic pitfall of ultrasound‑based bladder scanners in the context of intraperitoneal free fluid. The scanner estimated a bladder volume of 230 mL, which appeared inconsistent with the clinical picture of acute urinary retention and the subsequent drainage of 300 mL of bloody urine, alongside 1.5 L of intraperitoneal urinary extravasation at laparotomy. Abdominal fluid has been shown to be a confounding factor that limits the accuracy of bladder scanner measurements, reducing reliability in patients with low urine output or abnormal intra‑abdominal fluid states [9]. This underscores the need for cautious interpretation of bladder scanner readings in patients presenting with acute abdomen or suspected urinary tract injury.

Although CT cystography or conventional retrograde cystography are considered the gold‑standard investigations for diagnosing bladder rupture [5], they were not performed in this case because the patient required immediate operative management. As is standard in the evaluation of acute abdomen, an intravenous‑contrast CT scan was obtained first. The CT findings were sufficient to justify proceeding directly to emergency laparotomy, during which the diagnosis of intraperitoneal bladder rupture was confirmed.

Bladder ruptures typically occur at the bladder dome, the thinnest part of the bladder wall [5], consistent with our patient’s extensive intraperitoneal tear. Despite the 15 cm extent of the rupture, the presence of residual intravesical urine at catheterization is anatomically plausible, as partial apposition of the tear edges, intravesical clot formation, and surrounding peritoneal structures can transiently limit continuous leakage of urine from the bladder into the peritoneal cavity.

Surgical repair remains the standard treatment for intraperitoneal bladder rupture because of the high risk of peritonitis and sepsis [10]. Our patient’s full recovery following primary closure and drainage is consistent with recent case series, emphasizing that timely intervention yields favourable outcomes even in extensive rupture [7].

## Conclusions

SRUB is a rare but potentially life-threatening condition that often presents with non-specific symptoms, leading to delayed diagnosis. This case highlights the importance of early recognition of spontaneous bladder rupture in patients presenting with acute abdomen, particularly following binge alcohol intake, and underscores key diagnostic pitfalls, including unreliable bladder scanner measurements and pseudo-renal failure due to autodialysis.

When a concordant clinical picture is present, acute abdominal pain with urinary symptoms or oliguria, unexplained intraperitoneal free fluid, biochemical evidence consistent with urine autodialysis, or a history suggesting prolonged bladder overdistension (for example, recent binge alcohol consumption or use of bladder‑affecting medications), clinicians should maintain a heightened index of suspicion and pursue targeted assessments. Prompt surgical repair remains the definitive treatment for intraperitoneal rupture and is essential to reduce morbidity. Finally, this case raises a plausible role for antimuscarinic agents in permitting prolonged overdistension; a prospective study is needed to determine whether such medications are an independent, generalizable risk factor.
